# Carbon nano-onions (multi-layer fullerenes): chemistry and applications

**DOI:** 10.3762/bjnano.5.207

**Published:** 2014-11-04

**Authors:** Juergen Bartelmess, Silvia Giordani

**Affiliations:** 1 Istituto Italiano di Tecnologia, Nano Carbon Materials, Via Morego 30, 16163 Genova, Italy

**Keywords:** carbon nanomaterials, carbon nano-onions, fullerenes, functionalization

## Abstract

This review focuses on the development of multi-layer fullerenes, known as carbon nano-onions (CNOs). First, it briefly summarizes the most important synthetic pathways for their preparation and their properties and it gives the reader an update over new developments in the recent years. This is followed by a discussion of the published synthetic procedures for CNO functionalization, which are of major importance when elucidating future applications and addressing drawbacks for possible applications, such as poor solubility in common solvents. Finally, it gives an overview over the fields of application, in which CNO materials were successfully implemented.

## Review

### Introduction

Since the discovery of the fullerene C_60_ in 1985 by Curl, Kroto and Smalley [1], carbon nanomaterials have been the focus of interdisciplinary chemical research. In the following years, several other carbon based nanomaterials were discovered, namely carbon nanotubes (CNTs) [2–4], carbon nanohorns [5], nanodiamonds [6] and graphene [7]. Multi-shell fullerenes, known as carbon nano-onions (CNOs) and discovered by Ugarte in 1992 [8], are structured by concentric shells of carbon atoms. Over the last years, different methods for their synthesis have been developed and their properties have been widely studied. In addition, the chemical functionalization of CNOs has been investigated and several synthetic pathways were found to be applicable for the introduction of a variety of functional groups. Chemically modified CNOs were probed in different fields of application and have revealed to be a promising nanomaterial that attracts a growing interest among researchers and opens new avenues for investigation.

### Preparation and structural properties of carbon nano-onions

**Figure 1:**
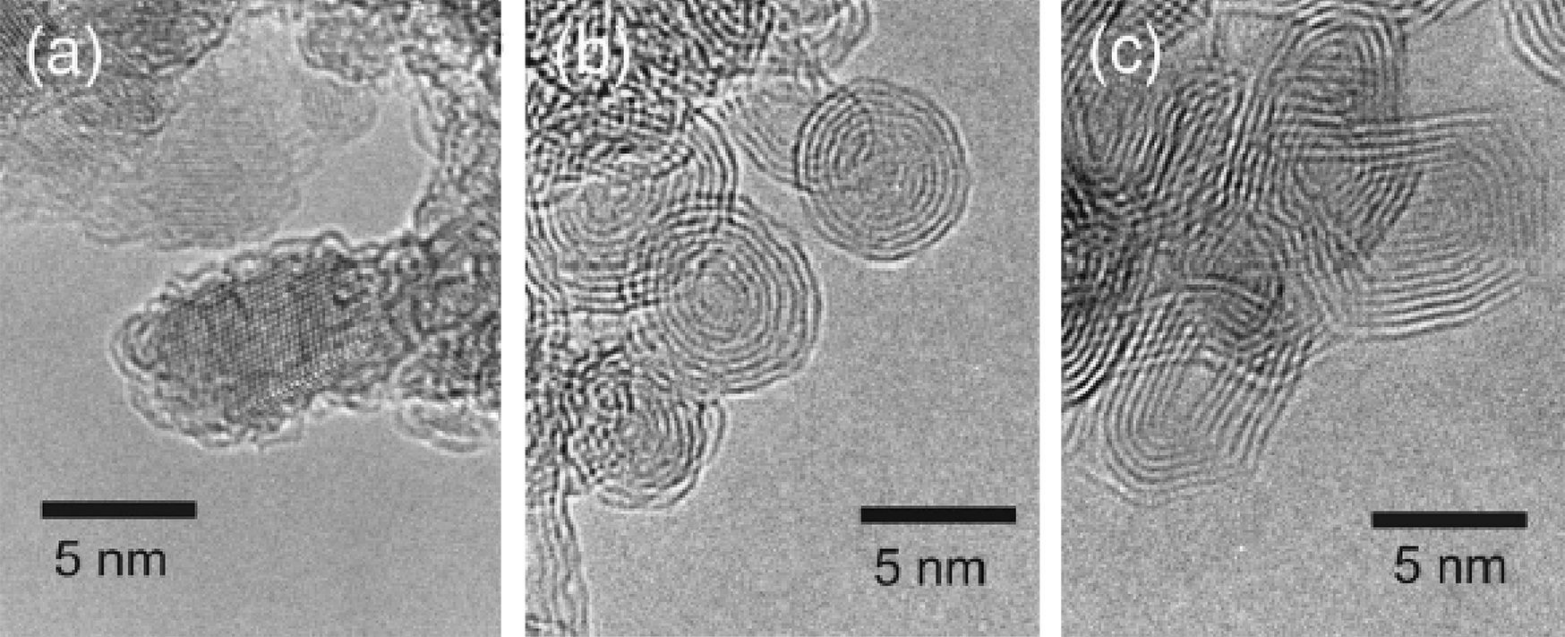
HRTEM images of (a) diamond nanoparticles, (b) spherical carbon onions, and (c) polyhedral carbon onions. Diamond nanoparticles are transformed into spherical onions at about 1700 °C. Polyhedral onions are dominant in the sample annealed above 1900 °C. Reprinted with permission from [12]. Copyright 2001 AIP Publishing LLC.

Carbon nano-onions were first discovered by Ugarte in 1992, who obtained them by intense electron irradiation of carbon soot [8]. CNOs were later found to be part of detonation soot [9], and a variety of different methods for their synthesis were reported. For a detailed review of the different methods and related mechanisms of CNO formation, we refer to the book chapter of Luis Echegoyen and co-workers [10]. A common method, for the preparation of small CNOs consisting of approx. 5–8 carbon shells, uses nanodiamonds as starting material. Nanodiamonds can be converted to graphitic CNOs by heat treatment (Figure 1) [11–12] or by electron radiation [13]. Another method is the formation of CNOs by arc discharge of graphite in liquids such as liquid nitrogen or water [14–15]. A recent novel method for the preparation of large CNOs (30 nm diameter) includes the use of inorganic starting material, such as CuCl_2_·2H_2_O and CaC [16]. Large CNOs with distinct fluorescence emission were produced from wood wool, a natural resource, which was pyrolized and then subsequently treated with concentrated nitric acid [17]. Nowadays, CNOs can be produced in gram-scale quantities by treatment of commercially available nanodiamonds, or by the combustion of naphthalene [18]. This good availability of different CNO materials, grants the future investigation of the applications of CNOs in a variety of fields.

The characteristic properties of CNOs render them of great interest for a large number of applications, as we will elucidate in the corresponding section of this review article. The diameter of the CNO nanomaterial depends on the synthetic protocol, but nevertheless, CNOs exhibit in general a high surface area to volume ratio. In his initial studies, Ugarte reported distances between the carbon layers of 0.34 nm, which is in good accordance to the distance of the layers in bulk graphite [8]. In a report from 1995, Daniel Ugarte refers to CNOs as onion-like graphitic particles, which display a wide range of structures, explicitly including polyhedral to nearly spherical morphologies in his definition of CNOs [19]. It is worth to mention, that in some reports the authors utilize the term onion-like carbons (OLCs), when referring to CNOs. In this review article, we have usually included the diameter of the utilized CNO nanomaterial, together with their fabrication method. If there are any divergent structural properties from the common definition of CNOs observed, we have included this information as well.

**Figure 2:**
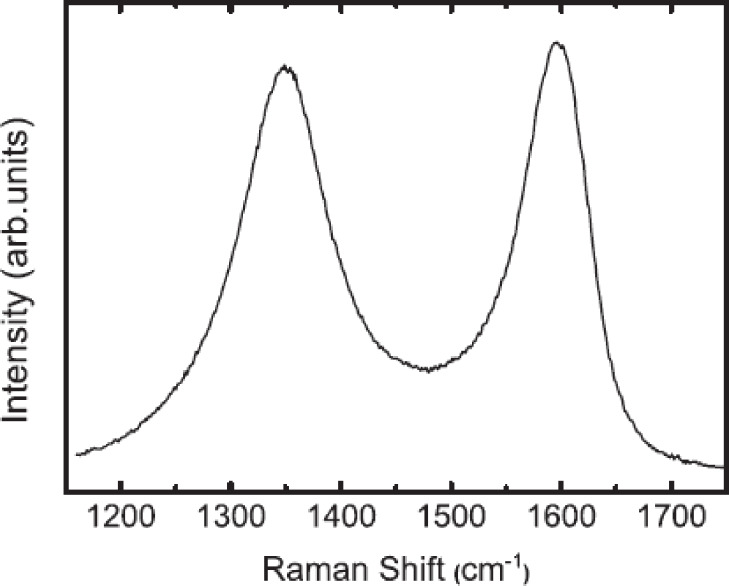
Typical Raman spectra of pristine CNOs. Reprinted with permission from [21]. Copyright 2013 Elsevier.

High-resolution transmission electron microscopy (HRTEM) has been widely employed to visualize CNOs and to study the mechanisms of CNO formation and their structural properties. Raman spectroscopy is another useful technique for the structural characterization of CNOs and corroborates the basic graphitic structure of carbon nano-onions [10,12,20–21]. Typically, two broad Raman bands can be readily observed in the area between 1300 and 1600 cm^−1^ (Figure 2). The D-band at around 1350 cm^−1^ resembles structural disorder due to the presence of sp^3^ carbons, while the G-band at around 1580 cm^−1^ corresponds to the E_2g_ mode of sp^2^-hybridized carbon frameworks. Covalent CNO functionalization usually leads to an increase of the D-band intensity, due to the increase of sp^3^-hybridized carbon atoms.

### Functionalization of carbon nano-onions

The access to soluble CNOs is important for different applications. Analogous to carbon nanotubes, CNOs display poor solubility in both aqueous and organic solvents. This is due to aggregation, promoted by strong intermolecular interactions such as van-der-Waals forces. To overcome this tendency to aggregate, functionalization of the surface of the carbon materials is the method of choice. The covalent as well as the non-covalent functionalization of CNTs [22–24] have been widely studied in the past decades and can serve as inspiration for possible synthetic strategies to decorate CNOs with a variety of functional groups and also to increase the solubility of CNO materials.

**Scheme 1:**
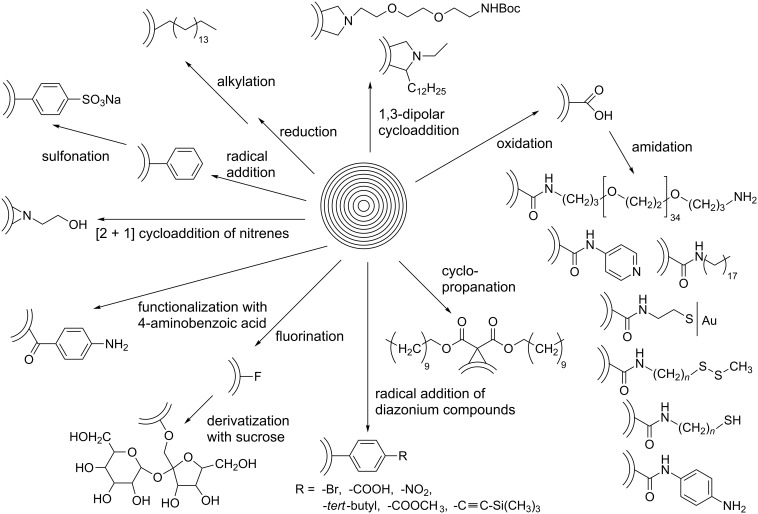
Covalent functionalization pathways for CNOs.

The following chapter summarizes the published literature regarding the reported methods for the covalent functionalization of CNOs (Scheme 1 and Table 1). In addition, we will give an overview over some CNO-containing composite materials. Except for some of these composites, the non-covalent functionalization of CNOs, especially with small molecules or surfactants, which is widely described for CNTs [23], has not been reported so far.

**Table 1:** Overview of covalent functionalization of CNOs.

reaction	description and added functional groups	reference

1,3-dipolar cycloaddition	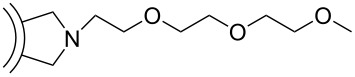	[25]
	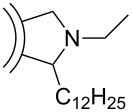	[26]
	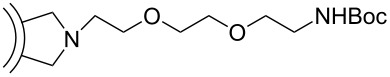 and subsequent amidation with ferrocene	[34]
oxidation	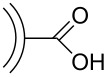	[26–27,36,59]
amidation of oxidized CNOs	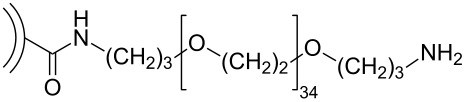	[26]
	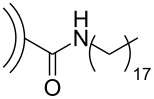	[26]
	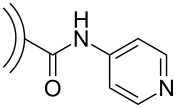 and subsequently Zn-tetraphenylporphyrin (ZnTPP)	[32]
	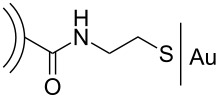 further CNO functionalization with biotinamidohexanoic acid and subsequently avidin	[36]
	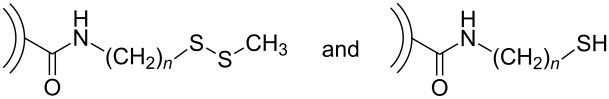	[83]
	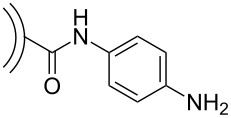 and subsequent polymerization with *p*-phenylenediamine	[45]
cyclopropanation	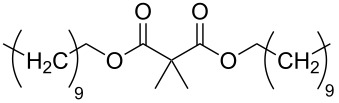	[27]
radical addition and subsequent sulfonation	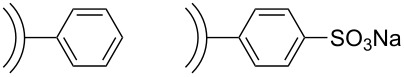	[27]
fluorination and subsequent derivatization with sucrose	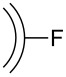	[28]
	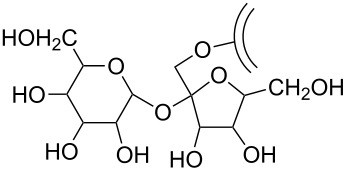	[29]
radical addition	bis-*o*-diynyl arene (BODA) co-polymers (Figure 2)	[30]
[2 + 1] cycloaddition of nitrenes	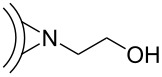 and subsequent polymerizations (Scheme 6)	[33]
radical addition of diazonium compounds	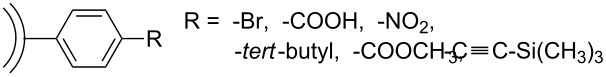 and subsequent Huisgen click-addition of Zn-porphyrin	[37]
reduction and alkylation	reduction with Na-K alloy followed by alkylation 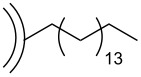	[42]
functionalization with 4-aminobenzoic acid	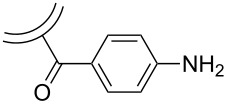 and subsequent polymerization with *p*-phenylenediamine	[45–46]

### Covalent functionalization

**Scheme 2:**
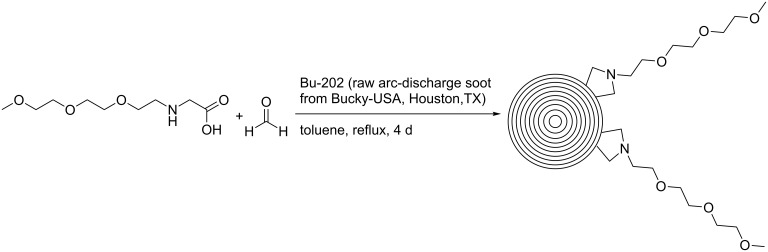
Covalent functionalization of CNOs by an azomethine ylide addition [25].

Synthetic procedures for the covalent functionalization of CNOs are largely based on previously described strategies for the functionalization of CNTs [22,24]. The first study reporting covalently functionalized CNOs was published in 2003, in which Prato et al. described an azomethine ylide addition reaction on CNOs [25] (Scheme 2). The CNO material was part of raw arc-discharge soot, which was processed as produced. The crude material was heated with an amino acid and paraformaldehyde. Following filtration and washing with toluene, the solid was suspended in chloroform. After one week, the formed precipitate was removed and the functionalized CNO material was obtained by evaporation of the chloroform. The authors reported that the final product was well soluble in common organic solvents such as ethanol, chloroform and dichloromethane. Evidence for a successful CNO functionalization was derived from ^1^H NMR spectroscopy, MALDI mass spectrometry and elemental analysis. TEM suggested that the individual CNOs had diameters between 60 and 300 nm. In addition, steady-state absorption and fluorescence spectroscopy was performed. It was found that the functionalized CNOs exhibit a distinct CNO-centered, excitation-wavelength-dependent fluorescence with a fluorescence quantum yield of 0.08. The fluorescence lifetimes depended on the emission wavelength and were determined to be 3.13 ns (at 550 nm) and 1.85 ns (at 400 nm) for the major component of a multiexponential fit. Transient absorption measurements indicated a strong difference of the absorption coefficients in the ground and excited state. However, some concerns were risen later, whether the investigated carbon nanomaterial in the present study was comparable to the CNOs described by Ugarte [26]. Especially since the authors report the distance between the internal shells of the CNOs to be ca. 4 nm, while Ugarte reported much smaller values of 0.34 nm.

**Scheme 3:**
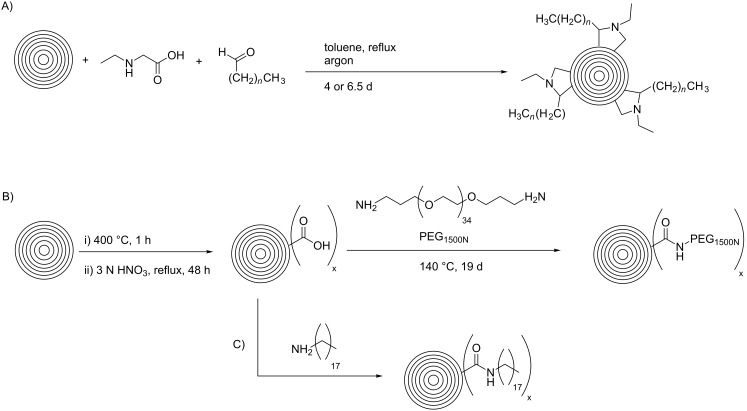
Methods for the covalent functionalization of CNOs by azomethine ylide addition on CNOs and amidation reactions of carboxylated CNOs [26].

Further functionalization strategies for CNOs were reported three years later by Echegoyen et al. [26], who described two novel methods for the functionalization of CNOs and, in addition, a variation of the mentioned 1,3-dipolar cycloaddition of an azomethine ylide (Scheme 3). The CNOs were prepared by arc discharge of graphite in water and had diameters of about 20 nm. For the 1,3-dipolar cycloaddition, the CNOs were purified by thermal annealing followed by heating under reflux in 3 N HNO_3_ and subsequent thermal annealing to remove potential organic functionalities. The reaction mixture, containing the purified CNOs, *N*-ethylglycine and dodecanal (or in another approach tridecanal) was refluxed, yielding the covalently functionalized CNO materials (Scheme 3A). The other two reactions presented in this study were carried out with purified carboxylated CNOs, which were fabricated from raw CNO material through thermal annealing and subsequent oxidation by reflux heating in 3 N HNO_3_. The first reaction utilizing oxidized CNOs was the functionalization with a diamine-terminated poly(ethylene glycol) (PEG_1500N_), realized by heating the CNOs in the PEG for 19 days (Scheme 3B). The second reaction was the functionalization of carboxylated CNOs with octadecylamine. In order to obtain this, the authors followed two different approaches. One was a solid state synthetic procedure, in which the CNOs were heated with 1-octadecylamine in an evacuated, sealed glass ampoule. The second approach was a microwave-assisted synthesis, in which CNOs and 1-octadecylamine were heated in DMF in a microwave reactor. Both approaches were successful and led to the desired octadecylated CNOs (Scheme 3C). To summarize, the synthetic pathways described in this study yielded functionalized CNO materials that were soluble in various organic solvents. One derivative, (CNO-PEG_1500N_), was also soluble in water.

**Scheme 4:**
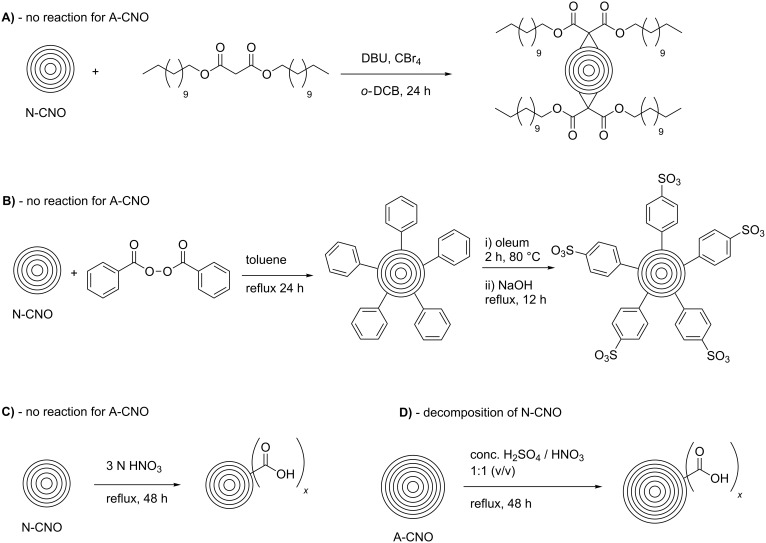
Comparison of the reactivity of small N-CNOs and larger A-CNOs, prepared by different methods [27].

In a comparative research work, Echegoyen et al. studied the reactivity of CNO materials of different sizes, which were prepared by different synthetic methods [27] (Scheme 4). A comparison was presented between larger and smaller CNOs. The former were produced by arc discharge of graphite under water (A-CNO), with a diameter of 15–20 nm and approx. 20–30 carbon shells while the latter were obtained by annealing nanodiamonds (N-CNO) and presented an average diameter of about 5 nm, having 6–8 carbon shells. The difference in diameter was found to be of great importance for the reactivity of the CNOs. The first reaction studied was a [2 + 1] Bingel–Hirsch cyclopropanation, which was successful only for the N-CNOs. The CNOs were reacted with a mixture of dodecyl malonate ester, carbon tetrabromide, and 1,8-diazabicyclo[5.4.0]undec-7-ene (DBU) in *o*-dichlorobenzene (Scheme 4A). The second reaction studied was a free-radical addition, in which the CNOs were heated under reflux with benzoyl peroxide as radical source in toluene. Also this reaction could be accomplished only with N-CNOs, while the A-CNOs did not react. The obtained phenylated CNOs were not soluble in organic solvents, but sulfonation with oleum, followed by treatment with aqueous NaOH lead to a highly soluble product, which formed stable dispersions in water as well as in ethanol (Scheme 4B). The last reaction investigated was based on an earlier reported oxidation of the N-CNOs by heating under reflux in 3 N HNO_3_ [26]. This treatment led to the introduction of carboxylic acid functionalities and caused, as verified by Raman spectroscopy, an increase of the number of defect sites on the CNO surface (Scheme 4C). In a similar procedure, A-CNOs were not oxidized. However, oxidation of A-CNOs was accomplished under much harsher conditions (conc. HNO_3_/H_2_SO_4_, 1:1 v/v, reflux) and led to comparable observations made for the oxidized N-CNO material. It is important to note that N-CNOs were completely destroyed under the harsh reaction conditions mentioned above (Scheme 4D). In conclusion, the authors found that the smaller N-CNO are much more accessible for covalent functionalization, while the functionalization of A-CNO requires an aggressive treatment. This observation is led back to a higher surface-to-volume ratio in the N-CNOs, compared to A-CNOs, as well as to a larger curvature increasing the degree of surface strain.

Another example for the functionalization of CNOs in a highly reactive environment was published by Khabashesku et al., who directly fluorinated CNOs under a stream of F_2_ and H_2_ [28]. CNO fluorination was carried out in a custom-built reactor with different fluorination temperatures. The CNOs used in this study were prepared from carbon black by heating and the formed individual CNOs had diameters between 50 and 100 nm. HF was generated in situ under the given reaction conditions in a continuous flow of a F_2_ gas mixture (10% F_2_/90% He) and H_2_. The successful fluorination led to an increase of the mass of the CNO material and the fluorinated CNOs (F-CNO) were well soluble in ethanol and other alcohols as well as in DMF. Characterization of the F-CNOs was carried out by a multitude of techniques such as FTIR, Raman and UV–vis spectroscopy, SEM/EDX, XRD, XPS, TGA and TEM. In addition, the authors reported that the fluorination of the F-CNOs was reversible upon treatment with hydrazine, which interestingly led also to a regeneration of the “broken” graphene layers of the CNO.

Some years later, based on the F-CNO material, the same group reported the preparation of water-soluble sucrose-functionalized CNOs [29]. In this case, F-CNOs were reacted with a lithium monosucrate derivative, which was previously synthesized from sucrose and lithium hydroxide. The sucrose-decorated CNOs showed an improved solubility of up to 200 mg·L^−1^ in water and 400 mg·L^−1^ in DMF.

**Figure 3:**
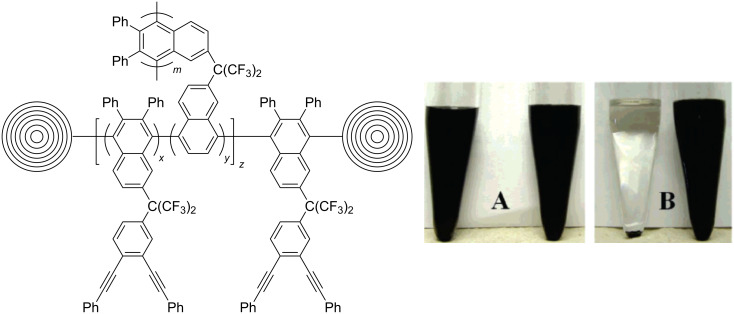
Structure of a CNO–BODA copolymer. (A) CNO starting material (left) and BODA-functionalized CNOs (right) suspended in NMP (0.67 mg·mL^−1^) immediately after sonication. (B) CNO starting material (left) and BODA-functionalized CNOs (right) suspended in NMP (0.67 mg·mL^−1^) after 2 h and 48 h of centrifugation. Reprinted with permission from [30]. Copyright 2007 American Chemical Society.

Smith et al. reported the first radical addition of a polymer to CNOs in 2007 [30]. The CNO starting material was prepared and purified following an earlier reported procedure [26], and then further functionalized with bis-*o*-diynyl arene (BODA), which is known to thermally form reactive bis-radicals [31]. After ultrasonication and heating of the CNOs and BODA in *N*-methyl-2-pyrrolidone (NMP) in a pressure vessel, a CNO–BODA copolymer was obtained. NMP suspensions of the CNO–BODA copolymer were found to be very stable, even at high concentrations of up to 0.67 mg·mL^−1^ (Figure 3). Characterization was carried out by a multitude of different techniques, such as GPC, TEM, TGA, XPS and Raman spectroscopy.

**Scheme 5:**
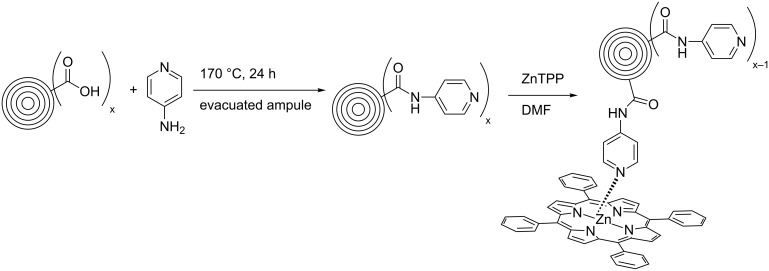
Preparation of pyridyl-CNOs and an illustration of their supramolecular interaction with Zn-tetraphenylporphyrin (ZnTPP) [32].

In 2008, Echegoyen et al. reported a first supramolecular CNO/Zn-porphyrin complex [32] (Scheme 5). In this set up, acid-treated CNOs bearing carboxylic acid functionalities, which were prepared by the oxidation of CNOs (diameter approx. 6 nm) with conc. HNO_3_/H_2_SO_4_ (3:1, v/v), were reacted with 4-aminopyridine in an amidation reaction. The synthesized water-soluble CNO material was characterized by using TEM, NMR, UV–vis and Raman spectroscopy. Based on TGA studies, the authors estimated that approximately one pyridine functionality per 120 CNO surface carbon atoms was present. The pyridine groups were then decorated with Zn-tetraphenylporphyrin (ZnTPP) what was confirmed by NMR spectroscopy and electrochemistry. No further spectroscopic studies were carried out with the presented CNO–ZnTPP supramolecular complex.

**Scheme 6:**
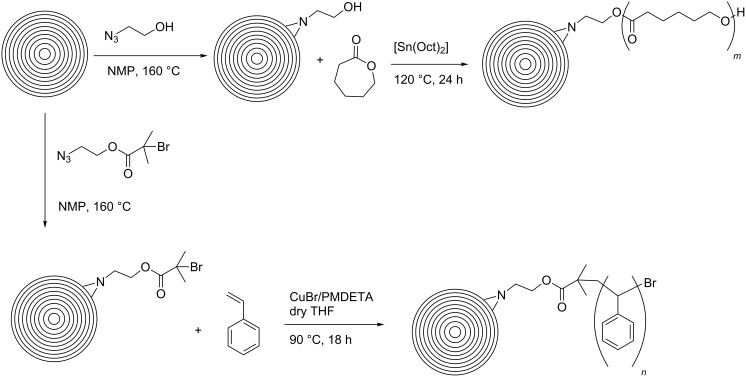
Illustration of polymerization reactions on CNOs following initial [2 + 1] cycloaddition reaction of nitrenes [33].

A further example of a [2 + 1] cycloaddition on CNOs was reported by Kong et al., who used different nitrene derivatives to covalently functionalize pristine CNOs [33] (Scheme 6). The CNO starting material was reacted with either 2-azidoethanol, leading to OH-functionalized CNOs (CNO-OH) or azidoethyl 2-bromo-2-methylpropanoate, yielding Br-functionalized CNOs (CNO-Br). The functionalized CNOs showed an increased solubility in organic solvents and water and were characterized by various techniques, such as TGA, XPS, TEM and Raman spectroscopy. Interestingly, the CNO-OH showed distinct fluorescence emission with an emission maximum at 453 nm in aqueous solution, while CNO-Br did not fluoresce. Based on these two CNO derivatives, the authors reported different polymerization reactions, where the CNOs served as macroinitiators. Firstly, CNO-OHs were used for a ring opening polymerization with ε-caprolactone in the presence of stannous octoate. In a second approach, CNO-Brs were decorated with polystyrene in an atom transfer radical polymerization reaction. Both polymer-functionalized CNO materials showed a good solubility in common organic solvents and give rise to potential future applications in various fields of technology.

The synthesis of ferrocene (Fc)-decorated CNOs was reported by Prato et al. [34]. CNOs were functionalized in a 1,3-dipolar cycloaddition reaction with a BOC-protected amin/amino acid [35] and paraformaldehyde. Subsequent deprotection of the amino functionality and reaction with Fc-carboxylic acid chloride lead to Fc-functionalized CNOs. By utilizing a TGA-based method for estimating the number of functionalizations per surface carbon atom of a CNO, the authors reported that the CNOs presented in this study contained one functional group per 36 surface carbon atoms. The properties of the Fc-CNO conjugates and the electronic interactions between the Fc and the CNO were investigated by electrochemical and spectroscopic methods, and supported by quantum chemical calculations.

A covalent functionalization of CNOs with biomolecules was reported by the groups of Plonska-Brzezinska, Simionescu and Echegoyen in 2010 [36]. In the first step, small CNOs (6–8 shells) were oxidized by using conc. H_2_SO_4_/HNO_3_ and subsequently functionalized with PEG to study their cytotoxicity on rat dermal fibroblasts. The result was that no significant cytotoxicity was observable, which renders this CNO material ideal for future biological applications. Toward the fabrication of CNO-biosensors, gold electrodes were initially decorated with a self-assembled monolayer of cysteamine on which the oxidized CNOs were deposited by an amidation reaction. In another amidation step, some of the unreacted carboxylic acid groups on the CNO surface were functionalized with biotin, which allows the attachment of biomolecules such as avidin. This first covalent functionalization of CNOs with biomolecules, promoted by biotin–avidin interactions, gives rise to future biological applications such as bio-sensors, especially since the authors of this study reported that the attached protein retains its biological activity.

**Scheme 7:**
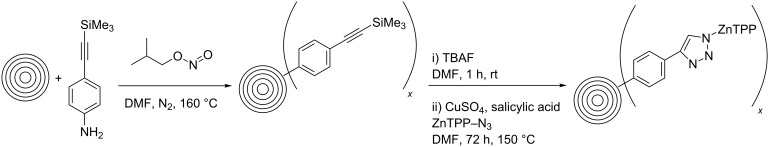
“Tour” functionalization of CNOs and subsequent “click”-addition of a ZnTPP-derivative [37].

A novel strategy for the surface functionalization of CNOs was published by our group in 2010 [37] (Scheme 7).

**Figure 4:**
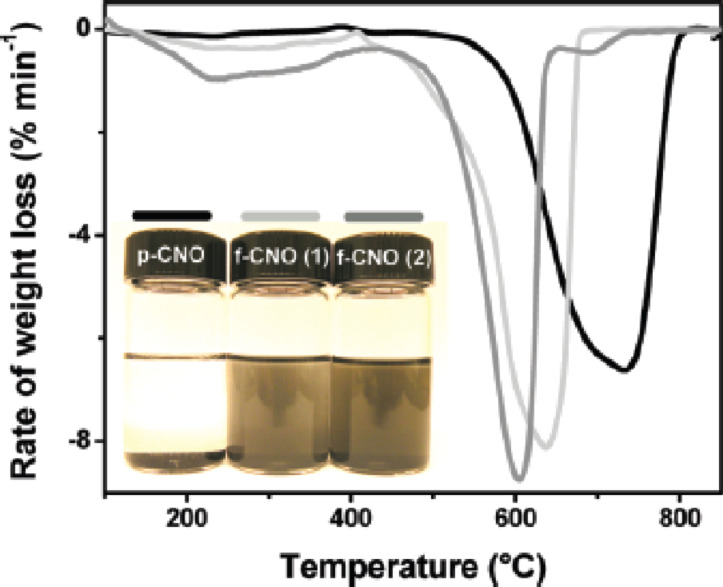
First derivative TGA weight-loss curves of pristine CNO (black), treated once (light gray) and treated three times with 4-bromoaniline (dark gray). Inset shows the enhanced CNO dispersibility in THF upon functionalization. Reprinted with permission from [37]. Copyright 2010 American Chemical Society.

The so-called Tour reaction is well studied for the covalent functionalization of carbon nanotubes by reacting them with in situ generated diazonium compounds [38]. This versatile reaction was used in the present study to attach different aniline derivatives to the surface of CNOs and thus introducing a variety of functional groups, such as bromides, benzoic acids, *tert*-butyl groups, nitro groups, methyl esters, and trimethylsilyl (TMS) acetylenes. For all reactions, the CNO starting material was suspended in DMF by ultrasonication and then the aniline derivative and isoamyl nitrite were subsequently added. After stirring, the different functionalized CNOs were obtained and characterized by Raman and TGA analysis. The Raman spectroscopy showed an increase of the D-band at 1354 cm^−1^, a clear confirmation of a successful covalent functionalization. In addition, TGA showed a significant weight loss at temperatures below 450 °C upon functionalization. Based on the method of Prato et al. [34], the number of surface carbon atoms per functionality was calculated to be between 22 and 60, depending on the different aniline derivatives used for functionalization. Multiple repetitions of the reaction lead to a further increase of the degree of functionalization (Figure 4). All kinds of functionalization led to an increased dispersibility of the CNOs. In a next step, the CNO–TMS acetylide material was first deprotected and then the free acetylene group was coupled with zinc triphenyl azidophenyl porphyrin in a copper-mediated “click” reaction. The successful functionalization was verified by TGA analysis as well as by Raman, absorption and fluorescence spectroscopy.

**Scheme 8:**
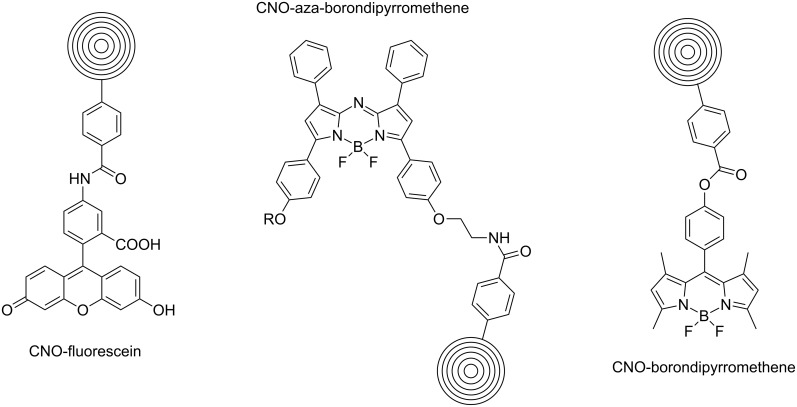
Fluorophore–CNO conjugates derived from benzoic acid-functionalized CNOs [39–41].

Following the aforementioned functionalization of CNOs with benzoic acid [37], fluoresceinamine-based fluorophores [39] as well as NIR-emitting aza-borondipyrromethenes (azaBODIPYs) were attached to the CNOs through an amidation reaction [40]. In another very recent study, we attached a *meso*-phenol-substituted borondipyrromethene (BODIPY) fluorophore on the same benzoic acid functionalized CNO nanomaterial through an esterification reaction [41]. These fluorescent-tagged CNOs (Scheme 8) were then used for in vitro fluorescence imaging, which will be discussed in the corresponding section of this review article.

Recently, Echegoyen et al. reported for the first time the alkylation of CNOs [42], which was achieved by a reductive process utilizing a Na–K alloy. CNOs were added to a previously prepared, deep-blue solution of the Na–K alloy in 1,2-dimethoxyethane under inert atmosphere affording a brownish dispersion. Then an excess of 1-bromohexadecane was added as electrophile and the alkylated CNO material (CNO-C_16_) could be recovered. TGA analysis and IR as well as Raman spectroscopy were used to verify the successful alkylation of the CNOs. It was reported that the CNO-C_16_ exhibits an outstanding solubility in a multitude of organic solvents, even in high concentrations of up to 0.1 mg·mL^−1^. This high solubility enabled the use of ^1^H NMR spectroscopy, which corroborated the presence of alkyl groups on the CNO surface. Additional HRTEM and SEM experiments were carried out to further support the successful functionalization and excellent solubility of CNO-C_16_. The authors also studied the reversibility of this alkylation reaction, which could be accomplished by annealing the CNO-C_16_ material at 415 °C, which was supported by Raman spectroscopy.

### CNO composites

**Scheme 9:**
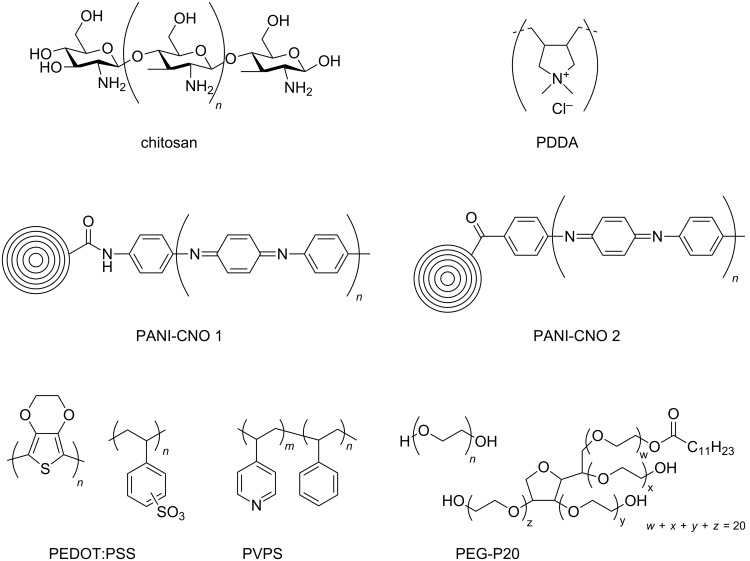
Schematic overview over the different polymeric structures utilized to functionalize CNOs [43–48].

In addition to the previously mentioned BODA-based CNO nanocomposites [30] and polymer-functionalized CNOs prepared by a [2 + 1] cycloaddition of nitrenes [33], several other CNO-containing composites have been reported (Scheme 9). In one study, composites consisting of CNOs and poly(diallyldimethylammonium chloride) (PDDA) or chitosan (Chit) were prepared and their electrochemical properties were studied [43]. In another study, CNO–PDDA composite films were used for the detection of dopamine in the presence of ascorbic acid and uric acid in solution [44]. The concentration of dopamine could be determined in a range between 5 × 10^−5^ and 4 × 10^−3^ mol·L^−1^. They also reported the in situ polymerization of aniline on phenylene amine-terminated CNO derivatives [45]. This polyaniline (PANI)-functionalized CNOs were characterized by a multitude of techniques and showed an excellent solubility in protic solvents, which gives rise to several future applications of this material. In a follow-up study, the properties of CNO–PANI composites were compared to other PANI-decorated carbon nanostructures [46]. In general, PANI films containing carbon nanostructures showed improved properties like a lower resistance and higher mechanical stability than pure PANI films. Another CNO-containing composite material was prepared from unmodified or oxidized CNOs and poly(3,4-ethylenedioxythiophene):poly(styrenesulfonate) (PEDOT:PSS) [47]. In a very recent study, they reported the non-covalent functionalization of CNOs with poly(4-vinylpyridine-*co*-styrene) (PVPS) and poly(ethylene glycol)/Polysorbate 20 (PEG/P20) [48]. The PVPS polymers were then further functionalized with thiols. Both CNO-containing polymers, could be modified with flavonoid compounds, for the example quercitin, a compound known for its anti-inflammatory potential [49], giving rise to future applications in nanomedicine.

Some additional metal oxide-containing composite materials were studied for applications as electrode materials in capacitors and lithium-ion batteries and will be discussed in the corresponding part of this review article.

### Toxicological aspects

In the context of applications in biology and medicine, newly employed nanomaterials should be carefully evaluated with regard to biocompatibility, environmental health and safety, secure processing and sustainable engineering.

In the case of CNOs, limited data regarding their biocompatibility has been published. An initial report, investigating the toxicity of large CNOs with a diameter of about 30 nm was published in 2005 by Chen et al. [50]. They probed the effects of large CNOs produced by an underwater carbon-arc discharge, as well as of multi-wall carbon nanotubes (MWCTNs) on human skin fibroblasts and found more adverse effects upon exposure to MWCNTs as compared to CNOs. However, CNOs were also found to cause negative effects on the studied cell cultures. The first report investigating the toxicity of small CNOs dates back to 2010, when Echegoyen et al. investigated the biocompatibility of PEGylated CNOs by exposing rat dermal fibroblasts to different CNO concentrations [37]. The authors could show almost 100% viability of cells for concentrations of 30 and 300 μg·mL^−1^, and a minor reduction of approx. 15% for 3,000 μg·mL^−1^. Thus, it follows that small CNOs are not cytotoxic and can be used safely for biological studies. In two other reports, highly oxidized CNOs, derived from pyrolized wood wool, were used for in vivo imaging of *Drosophila melanogaster*, *Escherichia coli* and *Caenorhabditis elegans* [17,51]. In both studies, no toxic effects of the water-soluble CNOs on the investigated organisms were observed.

We recently reported the weak inflammatory potential and low cytotoxicity in vitro and in vivo of CNOs and their ability to be up-taken by antigen-displaying cells. In our work, small benzoic acid functionalized CNOs were compared with similar functionalized single wall carbon nanotubes [39]. CNOs showed a lower inflammatory potential than CNTs and we demonstrated that chemical functionalization attenuates their inflammatory properties. This was evidenced by a reduced secretion of the inflammatory cytokine IL-1β, and a pronounced decrease in the recruitment of neutrophils and monocytes following injection into mice. Subsequently, in two recent studies, we investigated the effects of two different, fluorophore functionalized CNOs on HeLa Kyoto [40] and MCF-7 cells [41] and did not observe any significant cytotoxicity. Our results let us believe that CNOs are promising materials for biological and medicinal applications.

### Applications

In the following chapter we give an overview over the different applications of CNOs. We have selected representative studies from all areas of materials science, nano- and biotechnology and chemistry where CNOs have been successfully applied. The presented functionalization pathways usually led to a largely improved solubility of the CNO materials and thus to an enhanced processability and applicability. In addition, the introduction of functional groups and functionalities for specific applications further improved the usability of CNO materials. However, while other carbon nanostructures have drawn large attention in a variety of fields of applications, CNOs can still be considered as being a niche of the research on carbon nanostructures.

#### Biological and environmental applications

**Figure 5:**
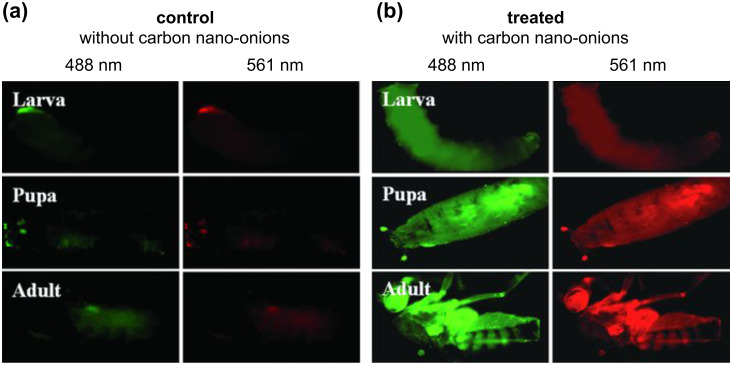
a) Autofluorescence images of different developmental stages of *Drosophila melanogaster* from larva to adult. b) *D. melanogaster* fed with water-soluble CNO, under 488 and 561 nm filters. Reprinted with permission from [17]. Copyright 2011 John Wiley and Sons.

**Biological imaging:** In contrast to other carbon nanomaterials such as CNTs [52] or carbon quantum dots [53], CNOs have not been widely employed in biological marking, yet. A first report was published in 2011 by Sarkar et al. by using large, defect rich CNOs, synthesized from wood waste, for imaging the life cycle of *D. melanogaster* (Figure 5) [17]. The authors claim that solubility in water was achieved by the presence of a large number of carboxylate groups on the CNO surface that originate from the production process. These carboxylate functional groups, together with the defective nature of the CNOs, also led to the observed fluorescence emission in the visible and NIR, which was imposed by spontaneous surface passivation and quantum confinement and allowed for multicolor biological imaging. The specimens were fed with fluorescent CNO nanomaterial that accumulated in the organisms and could be observed by fluorescence microscopy. Control experiments with non-CNO fed specimens were performed, excluding auto-fluorescence as reason for the observed luminescence of the organisms. Following their initial work describing in vivo biological marking of *D. melanogaster*, they used fluorescent CNOs also as imaging agents to study *E. coli* and *C. elegans* in vivo [51].

**Figure 6:**
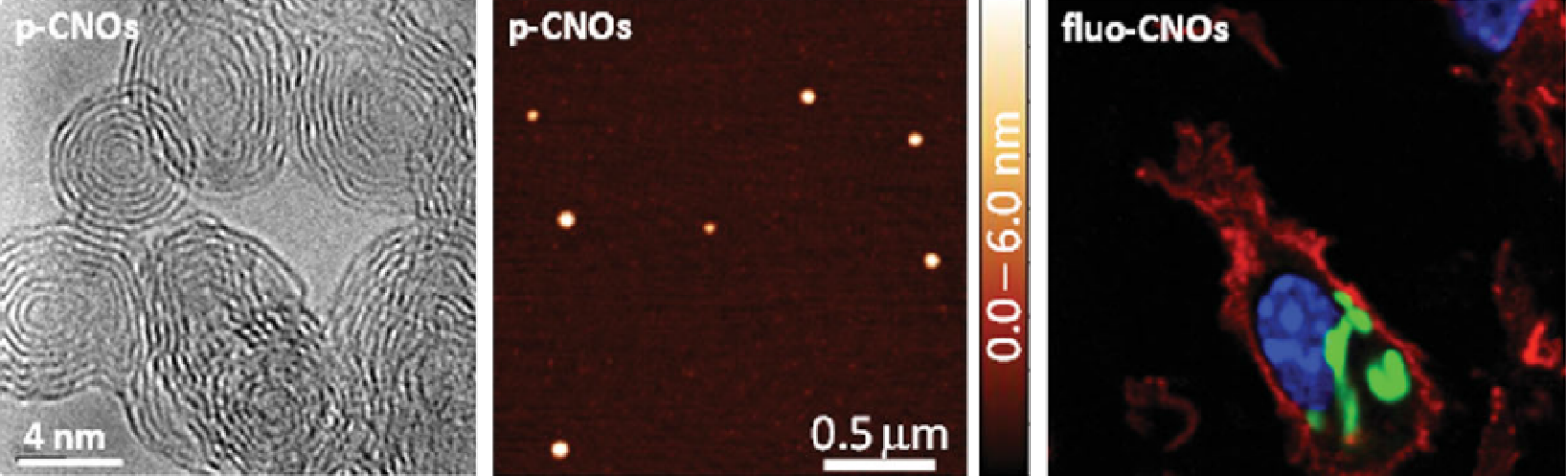
High-resolution TEM images of pristine CNOs (left). AFM topographs of pristine CNOs deposited on mica (center). Confocal microscopy of C57BL/6 BMDCs incubated in the presence of fluorescein-labelled-CNOs and stained with wheat germ agglutinin-Alexa Fluor594 (red), fluorescein (green) and nuclei stain Hoechst (blue) (right). Reprinted with permission from [39]. Copyright 2013 John Wiley and Sons.

In a recent report from our group [40], we used fluorescein-functionalized CNOs in a comparative toxicological study in vitro and in vivo, including biological marking (Figure 6). The cytotoxicity and immunomodulatory properties of the synthesized fluorescein-CNO derivative were elucidated and compared with similarly functionalized CNTs. We could show that CNOs exhibit efficient cellular uptake, weak inflammatory potential, and low cytotoxicity and are therefore promising material for biomedical applications.

**Figure 7:**
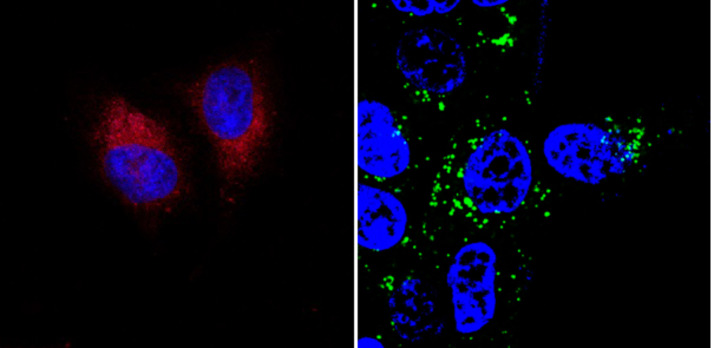
Confocal images of azaBODIPY-CNOs in HeLa Kyoto cells (left) and BODIPY-CNOs in MCF-7 cells (right). The blue luminescence is due to Hoechst 33342 nuclear stain. Reproduced with permission from [40] and [41]. Copyright 2014 The Royal Society of Chemistry.

Recently we demonstrated the cellular imaging of HeLa Kyoto [40] and MCF-7 cells [41] after incubating them with azaBODIPY- or BODIPY-functionalized CNOs (Scheme 8 and Figure 7). In both cases the CNO conjugates were readily internalized by the cells. In the earlier study, one of the azaBODIPY-CNO derivatives showed a pH-dependent switching (on–off) of the fluorescence, a feature that could also be observed inside the cells. The latter CNO nanomaterial was subject to co-localization experiments with Lysotracker Red dye and it was confirmed by high-resolution imaging that the CNOs were deposited in the lysosomes of the cells.

**Biological sensing:** In the aforementioned study of Luszczyn et al. [36], CNOs were covalently functionalized with biomolecules and studied for the first time as biosensors by using avitin–biotin interactions. The CNO served as linking layers between the biomolecules and the gold surface of the sensor and led to an amplified signal of the biosensor, as determined by surface plasmon resonance spectroscopy. In addition, the biocompatibility of CNOs was investigated and found to be excellent.

**Environmental remediation:** An application of CNO in environmental remediation was studied by Li group [54], who revealed that surface-oxidized CNO in aqueous suspensions have a high sorption capacity for heavy metal ions such as Pb^2+^, Cu^2+^, Cd^2+^, Ni^2+^ and Zn^2+^. The sorption capacity of oxidized CNOs was found to be up to ten times higher than the one of fullerene C_60_. These encouraging results could be a first step toward in situ remediation of heavy metal contaminants.

#### Electronic applications

**Figure 8:**
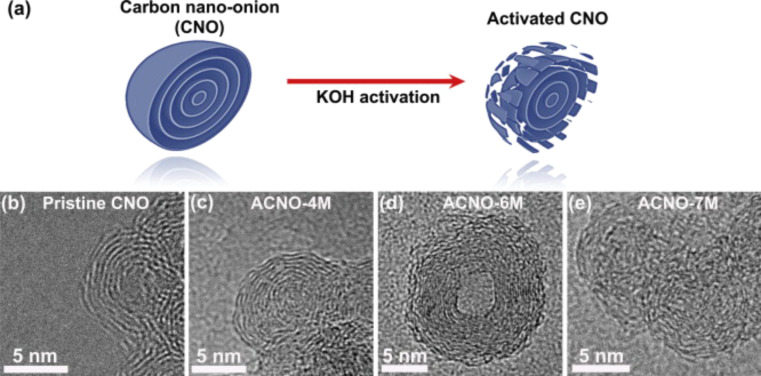
(a) A schematic showing the chemical activation of CNOs in KOH. TEM images of pristine CNO (b), ACNO-4M (c), ACNO-6M (d), and ACNO-7M (e). ‘‘ACNO-nM’’ denotes the activated CNO prepared using *n* mol·L^−1^ KOH solution. Reprinted with permission from [60]. Copyright 2013 Elsevier.

**Capacitors:** Carbon materials are commonly used as electrode materials in capacitors, but the first study probing CNOs as electrode materials in electrical double layer capacitors (EDLC) with an organic electrolyte was published only in 2007. The electrochemical performance of CNO electrodes was compared with electrodes made with nanodiamonds, multi-wall carbon nanotubes and carbon black [55]. Following this initial work, several groups studied CNO materials in supercapacitor electrodes. Bushueva et al. for example, found capacitance values of the investigated CNO material of 20–40 F·g^−1^ and 70–100 F·g^−1^ with acidic or basic electrolyte solutions, respectively [56]. In 2010, Pech et al. published the preparation and characterization of ultrahigh-power micrometer-sized supercapacitors based on CNOs [57]. In an extensive electrochemical study in different aqueous and organic electrolytes, McDonough et al. investigated the influence of the CNO structure on their electrochemical performance in supercapacitor electrodes [58]. The increase of the capacitance of CNO materials was the subject of two further studies. Borgohain et al. firstly oxidized the CNOs and subsequently functionalized the surface with polar carboxylic acid groups, which enabled them to precipitate RuO_2_ [59]. This functionalization led to an increase of the capacitance from 45 F·g^−1^ (for the pure CNO material) to 334 F·g^−1^ (for the RuO_2_·H_2_O–CNO composite material). Another strategy to increase the CNO capacitance is the activation of the CNO surface by treatment with 6 M KOH, creating porosity in the outer shells of the CNOs (Figure 8) [60]. The activated CNOs show largely improved properties compared to pristine CNOs with a maximum specific capacitance of 122 F·g^−1^ (vs 25.8 F·g^−1^), a power density of 153 kW·kg^−1^ (vs 123 kW·kg^−1^) and an energy density of 8.5 Wh·kg^−1^ (vs 1.5 Wh·kg^−1^).

Composite materials were studied for application in capacitors as well. The group of Echegoyen found that incorporation of CNOs in a Chit- or PDDA-polymer matrix leads to a large increase of the capacitance of the composite films (from 4–10 F·g^−1^ for the pure Chit or PDDA films to 20–30 F·g^−1^ for the corresponding CNO composite material) [43]. Also the specific electrochemical capacitance of a CNO–PANI composite (206.64 F·g^−1^) was much larger than for pure oxidized CNOs (12.15 F·g^−1^) [45]. After optimization in a follow-up study, the highest specific capacitance for a CNO–PANI composite was measured to be 525 F·g^−1^, which renders this composite material interesting for applications in supercapacitor electrodes [46]. Also the CNO/PEDOT:PSS composites, which were previously discussed, showed promising properties for the application as electrode material in supercapacitors, such as a specific capacity of 96 F·g^−1^, good cation-exchange properties and a simple synthesis [47]. In a recent study, the same group decorated the surface of CNO with Ni(OH)_2_ or NiO as pseudocapacitive redox material and showed that these composites can be promising materials for the development of supercapacitors [61]. In order to achieve this, the CNO surface was modified with nickel particles, which were synthesized in situ from nickel nitrate hexahydrate and ammoniumhydroxide in ethanol in the presence of (4-dimethylamino)pyridine (4-DMAP) as modifier in a one-pot multi-step reaction. Calcination of the CNO/4-DMAP/Ni(OH)_2_ composite led to the CNO/4-DMAP/NiO composite material. The electrochemical properties were promising, especially the specific electrochemical capacitance could be increased largely to 290.6 F·g^−1^ for the CNO/4-DMAP/NiO and 1225.2 F·g^−1^ for the CNO/4-DMAP/Ni(OH)_2_ composite, compared to pristine CNOs with 30.6 F·g^−1^.

Another example for CNO composite-based capacitors was reported by the group of H. Y. Yang [62]. The composite was prepared from KMnO_4_ and CNOs in different weight ratios in deionized water by heating in an autoclave. The formed CNO–MnO_2_ composite was then implemented in an asymmetric pseudocapacitor with the CNO–MnO_2_ composite as working electrode and nickel foam as counter electrode. The capacitance of pure MnO_2_ (40 F·g^−1^) could be increased by the incorporation of CNO up to 177.5 F·g^−1^. In addition, the authors report an excellent cycling stability with 99–101% retention of the specific capacitance after 1000 cycles.

**Figure 9:**
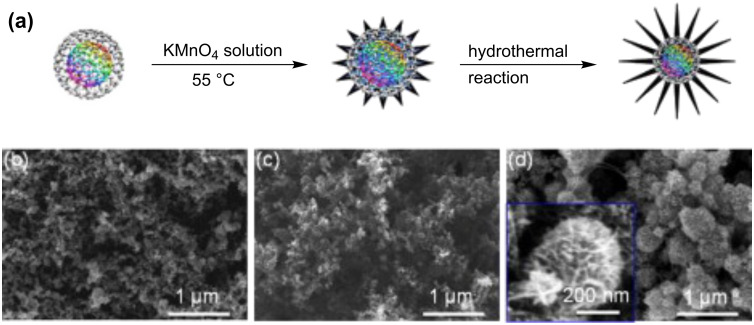
Note: The authors of the original report refer to CNOs as onion-like carbon (OLC) (a) Schematic diagram of the synthesis procedure of the core–leaf OLC–MnO_2_ hybrid nano-urchins. Scanning electron micrographs of (b) pure OLC, (c) intermediate product, and (d) final hybrid nano-urchins. Inset in (d) is the magnified image of a single urchin-like nano-architecture. Reprinted with permission from [65]. Copyright 2013 Elsevier.

**Lithium-Ion batteries:** Carbon nanotubes are widely studied for a use in lithium ion batteries [63]. However, also CNOs were studied for a potential application as anode materials. Han et al., for example, reported the large scale synthesis of CNOs starting from CuCl_2_·2 H_2_O and CaC_2_ and found that they exhibit a high capacity in combination with a promising cycling performance, which renders these as-prepared CNOs as potential anode materials for lithium-ion batteries [16]. However, no prototype batteries were prepared by the authors of this report. In two recent studies, H. Y. Yang and co-workers reported lithium-ion batteries incorporating CNOs in combination with Co_3_O_4_ [64] and MnO_2_ [65] as electrode material. In the earlier study, the CNO-containing anode material was prepared by a solvo-thermal method from cobalt acetate and CNOs and the authors found that the novel composite material showed improved electrochemical properties, compared to pristine Co_3_O_4_ electrodes. They observed, for example, an increase of the specific capacity from 190 mA·h·g^−1^ to 632 mA·h·g^−1^ at a current density of 200 mA·g^−1^ and also an increased rate capability [64]. In the latter report, the CNO hybrid material was prepared from KMnO_4_ and CNOs by a hydrothermal method (Figure 9) similar to the one the group reported earlier for the preparation of electrode materials for pseudocapacitors [62]. This material was then probed as anode material in lithium-ion batteries. It was found that the performance of pure MnO_2_ anodes could be significantly enhanced by the incorporation of CNOs. The specific capacity increased from 260 mA·h·g^−1^ to 630 mA·h·g^−1^, at a current density of 50 mA·g^−1^. In addition, the authors report an increased rate capability, stable cycling performance, and coulomb efficiency of nearly 100% [65].

**Fuel cells:** CNOs were also investigated as catalyst support for application in direct methanol fuel cells. For this, Xu et al. prepared CNOs decorated with Pt nanoparticles (Pt-CNO) and compared the performance of this novel catalyst material with common Pt/Vulcan XC-72 with encouraging results [66]. The novel Pt-CNO catalyst showed a higher surface area and smaller Pt particle size (3.05 nm vs 4.10 nm) than the reference system and the catalytic activity for the electro oxidation of methanol was increased by about 20%, rendering CNOs as a promising catalyst support for fuel cells.

**Terahertz-shielding:** In recent years, terahertz devices, circuits and terahertz-based communication systems have become important in many fields. This makes the development of materials for terahertz shielding essential, to limit electromagnetic interferences and thus reduce for example noise in cables and communication systems and signal coupling. Carbon materials have been widely studies for this application [67], including CNOs. The main research investigating the usability for CNOs for terahertz shielding was carried out by an international group of scientists and published in four papers in 2007 and 2008 [68–71]. After studying the terahertz absorption properties of CNO and CNO composites in a wide range of frequencies [68–70], the authors prepared CNO–poly(methyl methacrylate) (PMMA) composite films and studied the terahertz shielding properties in a frequency range of 0.1–3 THz and found a shielding of up to 14 dB [67,71].

#### Catalysis

One of the most important catalytical reactions in industry is the oxidative dehydrogenation (ODH) of ethylbenzene to styrene. In 2002, Keller et al. published a study showing the potential of CNOs as catalyst for this reaction with conversion levels of up to 92% after an activation period of 2 h and stable styrene yields of 62%, outperforming industrial K–Fe catalysts and other carbon materials [72]. In a later study, the groups of Su and Keller compared the catalytic behavior of nanodiamonds and CNOs for ODH catalysis [73]. Special attention was dedicated to the reactional behavior of sp^3^ and sp^2^ carbons of the catalyst surface as a function of time during catalysis. The authors conclude that the activity of the nanocarbon catalyst is largely influenced by the presence of oxygenated carbon species on the catalyst surface, which may eventually be formed during an initial period.

#### Tribology

In tribology, CNOs are widely studied and have shown promising results as lubricants. In 2002, Cabioc’h et al. reported that CNOs incorporated in silver layers significantly reduced wear, while the friction coefficient is largely unaffected by their presence [74]. Further studies investigated the use of CNOs as a solid state lubricant [75] and as an additive to the oil Krytox 143AB, where the aim was to improve the lubricating lifetime for space applications [76]. In the following years, several reports were published on the characterization of the tribological properties of CNO materials, produced through different methods [77–80]. Mechanisms by which CNOs can reduce friction and wear were investigated in greater detail in 2009 by Martin and collaborators in a combined experimental and computational study [81]. Computer simulations suggest that the lubrication of CNOs between two surfaces is caused by rolling–sliding of the CNO nanoparticles. These findings were corroborated by TEM observations of the wear particles and AFM imaging of the wear tracks.

#### Optical limiting

CNOs were also studied in optics and found to exhibit very efficient optical limiting [82]. Furthermore, the authors of this study compared the nonlinear optical response (upon pulsed laser excitation with 10 ns pulses, at 532 nm wavelength) of CNOs with nanodiamonds and concluded, that the optical response of CNOs is stronger than the one of nanodiamonds. In addition, the nonlinear optical refraction of the CNOs was found to be negligible. Finally, transient absorption studies of covalently functionalized CNOs yielded evidence for a strong difference of the absorption coefficients in the ground and excited state, which gives further rise to a possible application of this CNO material in optical limiting [25].

#### Molecular junctions in STM

In another recent report, the groups of Plonska-Brzezinska and Echegoyen prepared sulfide-terminated CNO derivatives, which can be used as molecular junctions in scanning tunneling microscopy (STM) [83]. This enabled the authors to study the conductivity of CNOs and compare their properties with comparable fullerene-C_60_ derivatives. The measured data suggests that the intrinsic conductivity of CNOs and C_60_ is within the same order of magnitude.

## Conclusion

Multi-shell fullerenes, known as carbon nano-onions (CNOs), were discovered in 1992 and are structured by concentric shells of carbon atoms in a graphitic interlayer distance. Analogous to carbon nanotubes, CNOs display poor solubility in both aqueous and organic solvents as well as a high surface area, compared to their volume. In order for their full potential to be realized, the solubility of the CNO nanomaterial has to be increased. To achieve this, a multitude of different synthetic pathways for the covalent surface functionalization has been reported. An alternative to the covalent functionalization is the surface decoration of CNOs with polymers or their incorporation into composites. CNOs have been implemented in different electronic applications, as electrode materials in capacitors, as anode materials in lithium-ion batteries, as catalyst support in fuel cells. They have even attracted the interest of NASA researchers for their tribological properties as additives for aerospace applications. Despite much interest in different carbon-based nano-materials, CNOs as functional constructs for intracellular transport have not been widely explored. However, given their size, homogeneity and purity (compared with carbon nanotubes) they could in principle add an important new avenue for the transport of imaging and therapeutic agents. These carbon particles have demonstrated high cellular uptake, low cytotoxicity and lower inflammatory potential than CNTs and a very promising future for biomedical applications.

## References

[1] Kroto H W, Heath J R, O’Brien S C, Curl R F, Smalley R E (1985). Nature.

[2] Bollmann W, Spreadborough J (1960). Nature.

[3] Iijima S (1991). Nature.

[4] Iijima S, Ichihashi T (1993). Nature.

[5] Iijima S, Yudasaka M, Yamada R, Bandow S, Suenaga K, Kokai F, Takahashi K (1999). Chem Phys Lett.

[6] Danilenko V V (2004). Phys Solid State.

[7] Novoselov K S, Geim A K, Morozov S V, Jiang D, Zhang Y, Grigorieva I V, Firsov A A (2004). Science.

[8] Ugarte D (1992). Nature.

[9] Kuznetsov V L, Chuvilin A L, Moroz E M, Kolomiichuk V N, Shaikhudtdinov S K, Butenko Y V, Mal’kov I Y (1994). Carbon.

[10] Echegoyen L, Ortiz A, Chaur M N, Palkar A J, Akasaka T, Wudl F, Nagase S (2010). Carbon Nano Onions. Chemistry of Nanocarbons.

[11] Kuznetsov V L, Chuvilin A L, Butenko Y V, Mal’kov I Y, Titov V M (1994). Chem Phys Lett.

[12] Tomita S, Sakurai T, Ohta H, Fujii M, Hayashi S (2001). J Chem Phys.

[13] Qin L-C, Iijima S (1996). Chem Phys Lett.

[14] Sano N, Wang H, Chhowalla M, Alexandrou I, Amaratunga G A J (2001). Nature.

[15] Alexandrou I, Wang H, Sano N, Amaratunga G A J (2004). J Chem Phys.

[16] Han F-D, Yao B, Bai Y-J (2011). J Phys Chem C.

[17] Ghosh M, Sonkar S K, Saxena M, Sarkar S (2011). Small.

[18] Choucair M, Stride J A (2012). Carbon.

[19] Ugarte D (1995). Carbon.

[20] Roy D, Chhowalla M, Wang H, Sano N, Alexandrou I, Clyne T W, Amaratunga G A J (2003). Chem Phys Lett.

[21] Krishnamurthy S, Butenko Y V, Dhanak V R, Hunt M R C, Šiller L (2013). Carbon.

[22] Singh P, Campidelli S, Giordani S, Bonifazi D, Bianco A, Prato M (2009). Chem Soc Rev.

[23] Herranz M A, Martin N, Guldi D M, Martin N (2010). Noncovalent Functionalization of Carbon Nanotubes. Carbon Nanotubes and Related Structures: Synthesis, Characterization, Functionalization, and Applications.

[24] Hauke F, Hirsch A, Guldi D M, Martin N (2010). Covalent Functionalization of Carbon Nanotubes. Carbon Nanotubes and Related Structures: Synthesis, Characterization, Functionalization, and Applications.

[25] Georgakilas N, Guldi D M, Signorini R, Bozio R, Prato M (2003). J Am Chem Soc.

[26] Rettenbacher A S, Elliott B, Hudson J S, Amirkhanian A, Echegoyen L (2006). Chem – Eur J.

[27] Palkar A, Melin F, Cardona C M, Elliott B, Naskar A K, Edie D D, Kumbhar A, Echegoyen L (2007). Chem – Asian J.

[28] Liu Y, Vander Wal R L, Khabashesku V N (2007). Chem Mater.

[29] Kuznetsov O V, Pulikkathara M X, Lobo R F M, Khabashesku V N (2010). Russ Chem Bull.

[30] Rettenbacher A S, Perpall M W, Echegoyen L, Hudson J, Smith D W (2007). Chem Mater.

[31] Bergman R G (1973). Acc Chem Res.

[32] Palkar A, Kumbhar A, Athans A J, Echegoyen L (2008). Chem Mater.

[33] Zhou L, Gao C, Zhu D, Xu W, Chen F F, Palkar A, Echegoyen L, Kong E S-W (2009). Chem – Eur J.

[34] Cioffi C T, Palkar A, Melin F, Kumbhar A, Echegoyen L, Melle-Franco M, Zerbetto F, Rahman G M A, Ehli C, Sgobba V (2009). Chem – Eur J.

[35] Kordatos K, Da Ros T, Bosi S, Vázquez E, Bergamin M, Cusan C, Pellarini F, Tomberli V, Baiti B, Pantarotte D (2001). J Org Chem.

[36] Luszczyn J, Plonska-Brzezinska M E, Palkar A, Dubis A T, Simionescu A, Simionescu D T, Kalska-Szostko B, Winkler K, Echegoyen L (2010). Chem – Eur J.

[37] Flavin K, Chaur M N, Echegoyen L, Giordani S (2010). Org Lett.

[38] Bahr J L, Yang J, Kosynkin D V, Bronikowski M J, Smalley R E, Tour J M (2001). J Am Chem Soc.

[39] Yang M, Flavin K, Kopf I, Radics G, Hearnden C H A, McManus G J, Moran B, Villalta-Cerdas A, Echegoyen L A, Giordani S (2013). Small.

[40] Giordani S, Bartelmess J, Frasconi M, Biondi I, Cheung S, Grossi M, Wu D, Echegoyen L, O’Shea D F (2014). J Mater Chem B.

[41] Bartelmess J, De Luca E, Signorelli A, Baldrighi M, Becce M, Brescia R, Nardone V, Parisini E, Echegoyen L, Pompa P P (2014). Nanoscale.

[42] Molina-Ontaria A, Chaur M N, Plonska-Brzezinska M E, Echegoyen L (2013). Chem Commun.

[43] Breczko J, Winkler K, Plonska-Brzezinska M E, Villalta-Cerdas A, Echegoyen L (2010). J Mater Chem.

[44] Breczko J, Plonska-Brzezinska M E, Echegoyen L (2012). Electrochim Acta.

[45] Plonska-Brzezinska M E, Mazurczyk J, Palys B, Breczko J, Lapinski A, Dubis A T, Echegoyen L (2012). Chem – Eur J.

[46] Plonska-Brzezinska M E, Breczko J, Palys B, Echegoyen L (2013). ChemPhysChem.

[47] Plonska-Brzezinska M E, Lewandowski M, Blaszyk M, Molina-Ontario A, Luciński T, Echegoyen L (2012). ChemPhysChem.

[48] Plonska-Brzezinska M E, Brus D M, Breczko J, Echegoyen L (2013). Chem – Eur J.

[49] Lei H, Luo J, Tong L, Peng L-q, Qi Y, Jia Z-g, Wei Q (2011). Food Chem.

[50] Ding L, Stilwell J, Zhang T, Elboudwarej O, Jiang H, Selegue J P, Cooke P A, Gray J W, Chen F F (2005). Nano Lett.

[51] Sonkar S K, Ghosh M, Roy M, Begum A, Sarkar S (2012). Mater Express.

[52] Gong H, Peng R, Liu Z (2013). Adv Drug Delivery Rev.

[53] Shen J, Zhu Y, Yang X, Li C (2012). Chem Commun.

[54] Seymour M B, Su C, Gao Y, Lu Y, Li Y (2012). J Nanopart Res.

[55] Portet C, Yushin G, Gogotsi Y (2007). Carbon.

[56] Bushueva E G, Galkin P S, Okotrub A V, Bulusheva L G, Gavrilov N N, Kuznetsov V L, Moiseekov S I (2008). Phys Status Solidi B.

[57] Pech D, Brunet M, Durou H, Huang P, Mochalin V, Gogotsi Y, Taberna T-L, Simon P (2010). Nat Nanotechnol.

[58] McDonough J K, Frolov A I, Presser V, Niu J, Miller C H, Ubieto T, Fedorov M V, Gogotsi Y (2012). Carbon.

[59] Borgohain R, Li J, Selegue J P, Cheng Y-T (2012). J Phys Chem C.

[60] Gao Y, Zhou Y S, Qian M, He X N, Redepenning J, Goodman P, Li H M, Jiang L, Lu Y F (2013). Carbon.

[61] Plonska-Brzezinska M E, Brus D M, Molina-Ontaria A, Echegoyen L (2013). RSC Adv.

[62] Wang Y, Yu S F, Sun C Y, Zhu T J, Yang H Y (2012). J Mater Chem.

[63] Landi B J, Ganter M J, Cress C D, DiLeo R A, Raffaelle R P (2009). Energy Environ Sci.

[64] Wang Y, Yan F, Liu S W, Tan A Y S, Song H, Sun X W, Yang H Y (2013). J Mater Chem A.

[65] Wang Y, Han Z J, Yu S F, Song R R, Song H H, Ostrikov K, Yang H Y (2013). Carbon.

[66] Xu B, Yang X, Wang X, Guo J, Liu X (2006). J Power Sources.

[67] Liu L, Das A, Megaridis C M (2014). Carbon.

[68] Shenderova O, Tyler T, Cunningham G, Ray M, Walsh J, Casulli M, Hens S, McGuire G, Kuznetsov V, Lipa S (2007). Diamond Relat Mater.

[69] Maksimenko S A, Rodionova V N, Slepyan G Y, Karpovich V A, Shenderova O, Walsh J, Kuznetsov V L, Mazov I N, Moseenkov S I, Okotrub A V (2007). Diamond Relat Mater.

[70] Shenderova O, Grishko V, Cunningham G, Moseekov S, McGuire G, Kuznetsov V (2008). Diamond Relat Mater.

[71] Macutkevic J, Adomavicius R, Krotkus A, Seliuta D, Valusis G, Maksimenko S, Kuzhir P, Batrakov K, Kuznetsov V, Moseenkov S (2008). Diamond Relat Mater.

[72] Keller N, Maksimova N I, Roddatis V V, Schur M, Mestl G, Butenko Y V, Kuznetsov V L, Schlögl R (2002). Angew Chem, Int Ed.

[73] Su D, Maksimova N I, Mestl G, Kuznetsov V L, Keller V, Schlögl R, Keller N (2007). Carbon.

[74] Cabioc’h T, Thune E, Rivière J P, Camelio S, Girard J C, Guérin P, Jaouen M, Henrard L, Lambin P (2002). J Appl Phys.

[75] Hirata A, Igarashi M, Kaito T (2004). Tribol Int.

[76] Street K W, Marchetti M, Vander Wal R L, Tomasek A J (2004). Tribol Lett.

[77] Matsumoto N, Joly-Pottuz L, Kinoshita H, Ohmae N (2007). Diamond Relat Mater.

[78] Joly-Pottuz L, Vacher B, Ohmae N, Martin J M, Epicier T (2008). Tribol Lett.

[79] Yao Y, Wang X, Guo J, Yang X, Xu B (2008). Mater Lett.

[80] Joly-Pottuz L, Matsumoto N, Kinoshita H, Vacher B, Belin M, Montagnac G, Martin J M, Ohmae N (2008). Tribol Int.

[81] Joly-Pottuz L, Bucholz E W, Matsumoto N, Phillpot S R, Sinnott S B, Ohmae N, Martin J M (2010). Tribol Lett.

[82] Koudoumas E, Kokkinaki O, Konstantaki M, Couris S, Korovin S, Detkov P, Kuznetsov V, Pimenov S, Pustovoi V (2002). Chem Phys Lett.

[83] Sek S, Breczko J, Plonska-Brzezinska M E, Wilczewska A Z, Echegoyen L (2013). ChemPhysChem.

